# HMGB4 is expressed by neuronal cells and affects the expression of genes involved in neural differentiation

**DOI:** 10.1038/srep32960

**Published:** 2016-09-09

**Authors:** Ari Rouhiainen, Xiang Zhao, Päivi Vanttola, Kui Qian, Evgeny Kulesskiy, Juha Kuja-Panula, Kathleen Gransalke, Mikaela Grönholm, Emmanual Unni, Marvin Meistrich, Li Tian, Petri Auvinen, Heikki Rauvala

**Affiliations:** 1 Neuroscience center, University of Helsinki, Finland; 2 Department of Biosciences, University of Helsinki, Finland; 3 Schools of Pharmacy and Medicine, Department of Bioengineering and Therapeutic Sciences, University of California, San Francisco, California, USA; 4 Institute of Biotechnology, University of Helsinki, Finland; 5 Institute for Molecular Medicine Finland, FIMM, University of Helsinki, Finland; 6 Department of Biochemistry, University of Maryland School of Medicine, Baltimore, Maryland, USA; 7 Department of Experimental Radiation Oncology, Division of Radiation Oncology, MD Anderson Cancer Center, Houston, Texas, USA; 8 Psychiatry Research Center, Beijing Hui Long Guan Hospital, Peking University, Beijing, China

## Abstract

HMGB4 is a new member in the family of HMGB proteins that has been characterized in sperm cells, but little is known about its functions in somatic cells. Here we show that HMGB4 and the highly similar rat Transition Protein 4 (HMGB4L1) are expressed in neuronal cells. Both proteins had slow mobility in nucleus of living NIH-3T3 cells. They interacted with histones and their differential expression in transformed cells of the nervous system altered the post-translational modification statuses of histones *in vitro*. Overexpression of HMGB4 in HEK 293T cells made cells more susceptible to cell death induced by topoisomerase inhibitors in an oncology drug screening array and altered variant composition of histone H3. HMGB4 regulated over 800 genes in HEK 293T cells with a p-value ≤0.013 (n = 3) in a microarray analysis and displayed strongest association with adhesion and histone H2A –processes. In neuronal and transformed cells HMGB4 regulated the expression of an oligodendrocyte marker gene PPP1R14a and other neuronal differentiation marker genes. In conclusion, our data suggests that HMGB4 is a factor that regulates chromatin and expression of neuronal differentiation markers.

HMGB-proteins are less than 30 kDa in size and bind to DNA in a manner that is dependent on the structure of the DNA and mainly independent of the sequence of the DNA^1^. HMGB1, -2 and -3 proteins are widely expressed in eukaryotes and highly conserved between species. They are composed of two DNA-binding HMGB-boxes and an acidic tail. HMGB4 is a mammalian specific protein that has two HMGB-boxes but lacks the acidic tail. Its nucleotide sequence is less conserved than the sequences of the other *HMGB* genes^2^.

Unlike other HMGB-proteins, HMGB4 has a very restricted expression pattern with extremely low expression levels in somatic cells. HMGB4 mRNA is undetectable during mouse embryonal development at the E10.5 stage but it is expressed in the brain and the pancreas at E12.5 and E14 stages, respectively^3^,^4^,^5^. During adulthood, the highest HMGB4 expression levels are observed in testes in round spermatids, elongating spermatids and in spermatocytes^6^. At the chromosomal level it localizes to transcriptionally inactive sex bodies of pachytene spermatocytes^6^. Lower expression levels of HMGB4 during adulthood are seen in the kidney and in the brain^2^. In rats the closely related protein Transition Protein 4 (TP4) is believed to be exclusively found in nuclei of elongating spermatids^7^. The regulation mechanism of the *HMGB4* gene is still mainly uncharacterized. In spermatozoa an active histone methylation mark, histone H3 dimethylated lysine 4, is present in the HMGB4 promoter indicating active transcription in haploid sperm cells^8^.

The functional role of endogenous HMGB4 in somatic cells is poorly understood. Previous studies showed that it is downregulated during neurosphere differentiation^5^. In canine osteosarcomas the expression of HMGB4 correlates with favorable prognosis and alterations in the HMGB4 gene region are associated with reoccurrence and death in melanoma^9^,^10^. Ectopic expression of HMGB4 in transformed cells represses transcription, inhibits cancer cell growth via the retinoblastoma dependent pathway, and potentiates the anti-cancer effects of both γ-ray irradiation and cisplatin^2^,^11^,^12^. Polymorphism in the human HMGB4 gene region have been associated with psychiatric disorders like ADHD and schizophrenia^13^,^14^. Further, HMGB4 expression in the mouse hippocampus is regulated by antidepressants, and in humans HMGB4 polymorphisms correlate with different antidepressant responses^15^,^16^.

Since HMGB4 is expressed during embryonal development and regulates growth of transformed cells, we have studied HMGB4 expression and cellular functions regulated by HMGB4 using transformed cell and developing brain cell models.

## Results

**Figure 1:**
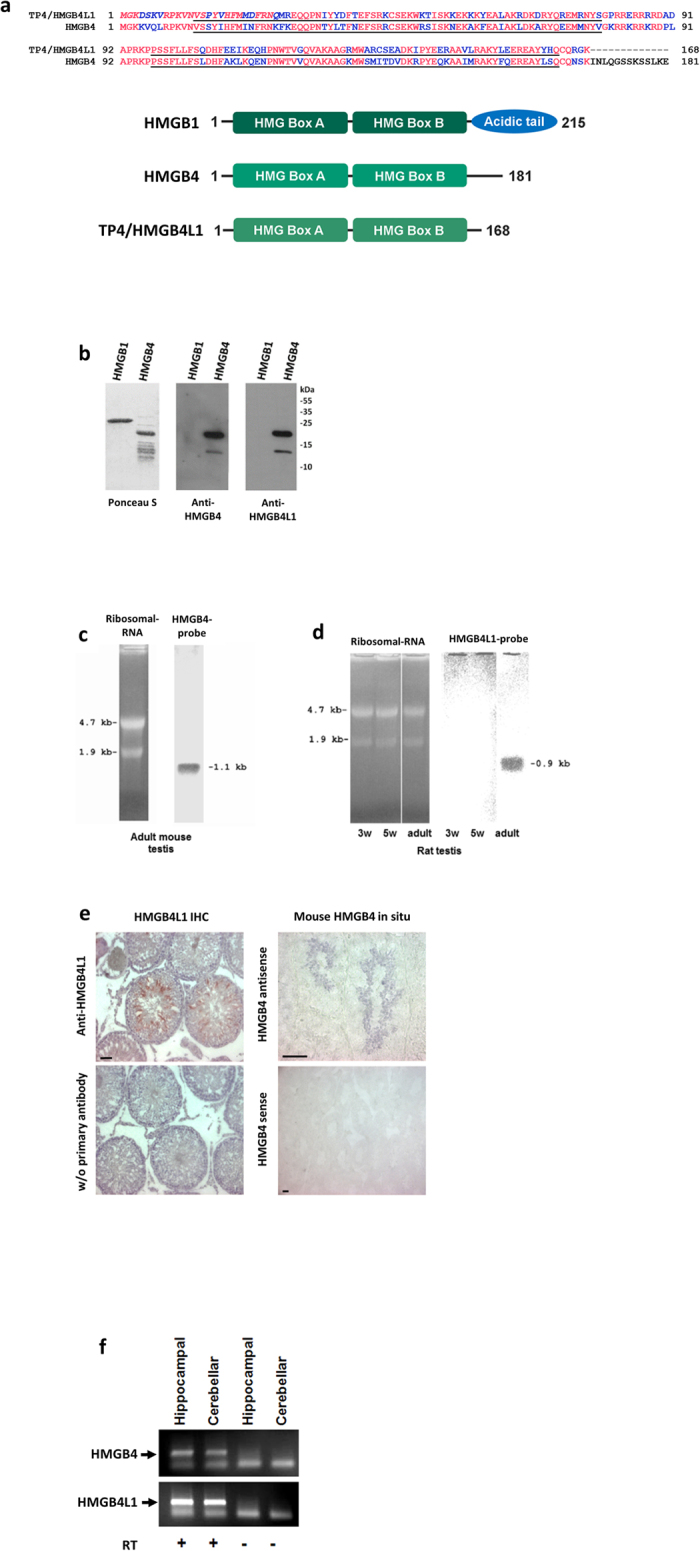
Characterization of HMGB4 and HMGB4L1. (**a**) Alignment of rat TP4/HMGB4L1 and HMGB4 amino acid sequences. HMGB-boxes A and B are underlined. Red letters indicate identical amino acids. An alternative allele in position 34 of TP4/HMGB4L1 is either a tyrosine or a isoleucine. Amino acids marked in italics have been identified by amino terminal amino acid sequencing^7^. Domain structures and number of amino acids of rat HMGB1, HMGB4 and TP4/HMGB4L1 are shown in the schematic picture. (**b**) Western –blot of recombinant mouse HMGB4. Recombinant HMGB4 was detected with Ponceau S –staining and with anti-HMGB4 and anti-HMGB4L1 antibodies. The antibodies did not detect recombinant HMGB1. (**c**) Northern Blot -analysis of the mouse HMGB4 transcript. Total RNA samples were isolated from adult mouse testes and analyzed via Northern Blot, using a probe derived from the entire coding sequence of mouse HMGB4. The probe detected a 1.1 kb band. Ethidum bromide stained ribosomal RNA is shown. (**d**) Northern Blot -analysis of rat HMGB4L1 transcript. Total RNA samples were isolated from adult rat testes and from developing rat testes, and analyzed via Northern Blot, using a probe derived from the entire coding sequence of rat HMGB4L1. The probe detected a 0.9 kb band in samples derived from testes of sexually mature rat. Ethidum bromide stained ribosomal RNA is shown. 3 w = 3 week old rat, 5 w = 5 week old rat. (**e**) Immunohistochemical staining of rat HMGB4L1 protein and *in situ* hybridization of mouse HMGB4 mRNA in adult testes. Anti-HMGB4L1 polyclonal antibody staining revealed intense HMGB4L1 expression in elongated spermatids (red). Haematoxylin was used as a counterstain. Control sections were stained without the primary antibody. HMGB4 mRNA localized to round and elongated spermatids. The control section shows adult mouse testes incubated with the sense probe. Bars represent 50 μm. (**f**) Expression of HMGB4 and HMGB4L1 mRNA in cultured rat neurons. Neuronal cells from the hippocampus and the cerebellum were cultured, and both HMGB4 and HMGB4L1 expression (arrows) was detected with RT-PCR. +RT =  reverse transcribed, -RT =  without reverse transcription.

Database searches revealed that both human and mouse have a single copy of the HMGB4-gene^2^,^17^, whereas the rat has two HMGB4-like genes, one on chromosome 5 and another one on the X-chromosome [coding for proteins HMGB4 (NP_001102933) and predicted high mobility group protein B4 –like (XP_006227590), respectively, in the NCBI database]. The predicted protein coded by the gene on the rat X-chromosome was identified as TP4^7^ (accession number AAB24466). The amino acid sequences of the rat HMGB4 and TP4 are 66% identical and 82% similar (Fig. 1a refs 2 and 7). TP4 is renamed high mobility group box 4 protein –like 1 (HMGB4L1) in this study. Comparison of HMGB4 and HMGB4L1 to the amino acid sequence of the archetype of the HMGB-proteins, HMGB1, revealed that rat HMGB4 is 42% identical and 67% similar, and HMGB4L1 is 43% identical and 66% similar.

A mouse recombinant HMGB4 protein is recognized by both anti-HMGB4 and anti-HMGB4L1 antibodies (Fig. 1b) suggesting immunogenic similarity. Both HMGB4 and HMGB4L1 are highly expressed in adult rat testes and coded by transcripts of approximately 1 kb size (Fig. 1c,d). Their expression occurs in elongated spermatids (Fig. 1e, refs 2,7,18), in brain (Supplementary Figure S2) and in neuronal cells (Fig. 1f).

**Figure 2:**
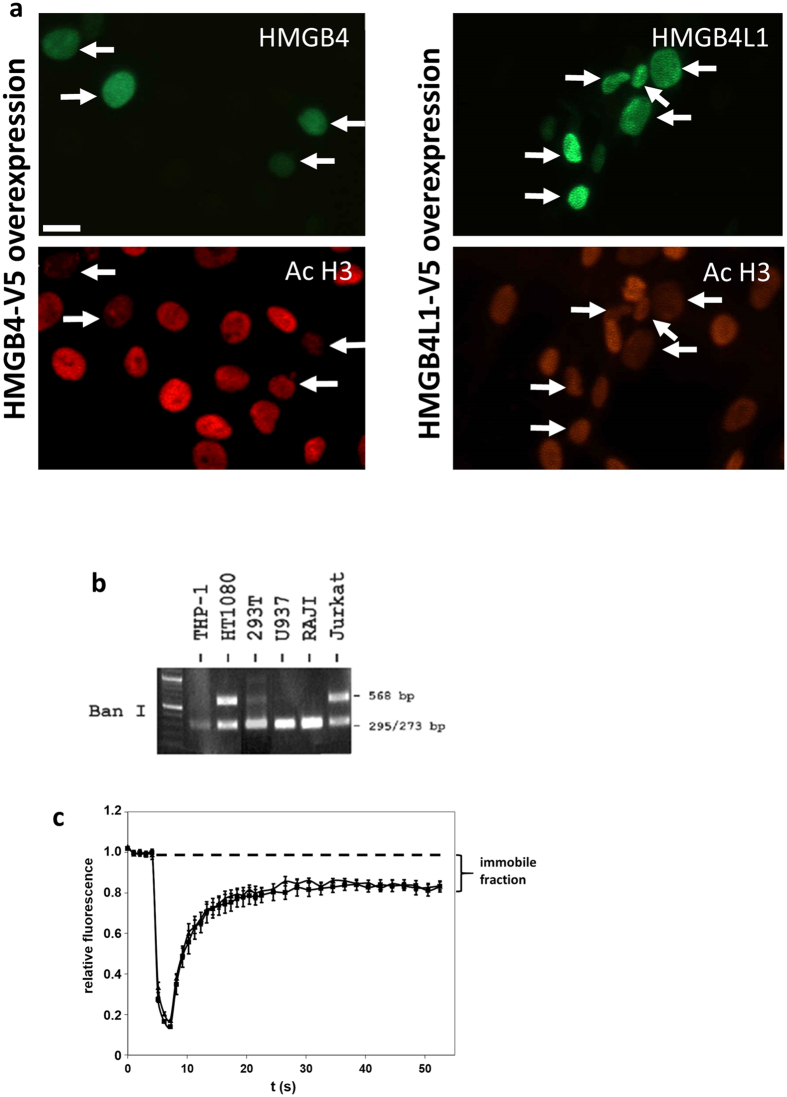
Nuclear localization of HMGB4 and HMGB4L1. (**a**) Rat glioblastoma C6 –cells transiently transfected with V5-tagged HMGB4 and HMGB4L1 were double immunostained with anti-V5-tag and anti-acetylated K9/K14 histone H3 antibodies. Immunofluorescence staining intensity of anti-acetylated K9/K14 histone H3 is reduced in C6 –cells transiently expressing HMGB4 or HMGB4L1. Arrows indicate HMGB4- and HMGB4L1-positive nuclei in double immunostained cells. Scale bar 20 = μm. (**b**) Single nucleotide polymorphism of the human HMGB4 gene. Genomic DNA of different human cell lines was amplified in PCR with primers specific to HMGB4 gene and subjected to Ban 1 restriction enzyme analysis. SNP rs10379 destroys Ban 1 cleavage site in the coding sequence of HMGB4 gene. Gel figure indicates that HT1080 and Jurkat cells are heterozygotes for rs10379 SNP. (**c**) Nuclear mobility FRAP analyses of wild type HMGB4 and rs10379 polymorphic human HMGB4 protein forms. NIH-3T3 -cells were transfected with an expression vector coding for the prominent allele or the polymorphic allele of human HMGB4-EGFP fusion proteins. One brightly fluorescent area in the nucleus with high expression of HMGB4-EGFP was bleached with three laser pulses and recovery of fluorescence was measured. Values from pre-bleach areas were determined as 1 and normalized values for bleached areas were calculated. The failing of full fluorescent recovery indicates the existence of an immobile fraction of HMGB4-EGFP in the nucleus. Triangles = wild type HMGB4, squares = SNP form of HMGB4; n = 5; ± SD.

We studied the functions of HMGB4 and HMGB4L1 using different cultured cell models. Similarly to HMGB4, HMGB4L1 localizes to the nucleus of cultured C6 cells (Fig. 2a). The mobility of EGFP –tagged HMGB –proteins in NIH-3T3 –cell nuclei was studied by the Fluorescence Recovery After Photobleaching (FRAP) assay using method described previously for the same cell line, which ectopically expresses HMGB –fusion proteins^19^. Half of the nucleus was photobleached and fluorescence recovery time was monitored. HMGB4-EGFP and HMGB4L1-EGFP had a similar mobility within the nucleus [half-time (t_1/2_) 5.3 ± 2.8 s and 8.3 ± 1.6 s, respectively]. Compared to HMGB4-EGFP and HMGB4L1-EGFP, the HMGB1-EGFP displayed a clearly higher mobility, with a t_1/2_ of 1.8 ± 0.5 s that is similar to t_1/2_ of HMGB1 as described by others^19^,^20^. A human HMGB4 nonsynonymous polymorph rs10379 was detected with restriction enzyme analysis and the mobility of proteins coded by the prominent allele and by the polymorphic allele was analyzed by another FRAP assay (Fig. 2b,c). In this particular assay bleaching and monitoring of fluorescence recovery was focused to one bright nuclear area, smaller than half of the nucleus, to obtain increased resolution. There was no difference in mobility between the forms coded by the prominent allele or by the polymorphic allele. However, fluorescence suggested existence of an immobile HMGB4-EGFP fraction that had not recovered during the time period observed. These results show that the two closely related proteins, HMGB4 and HMGB4L1, bind tightly to nuclei of living cells in contrast to HMGB1, which binds much more weakly.

**Figure 3:**
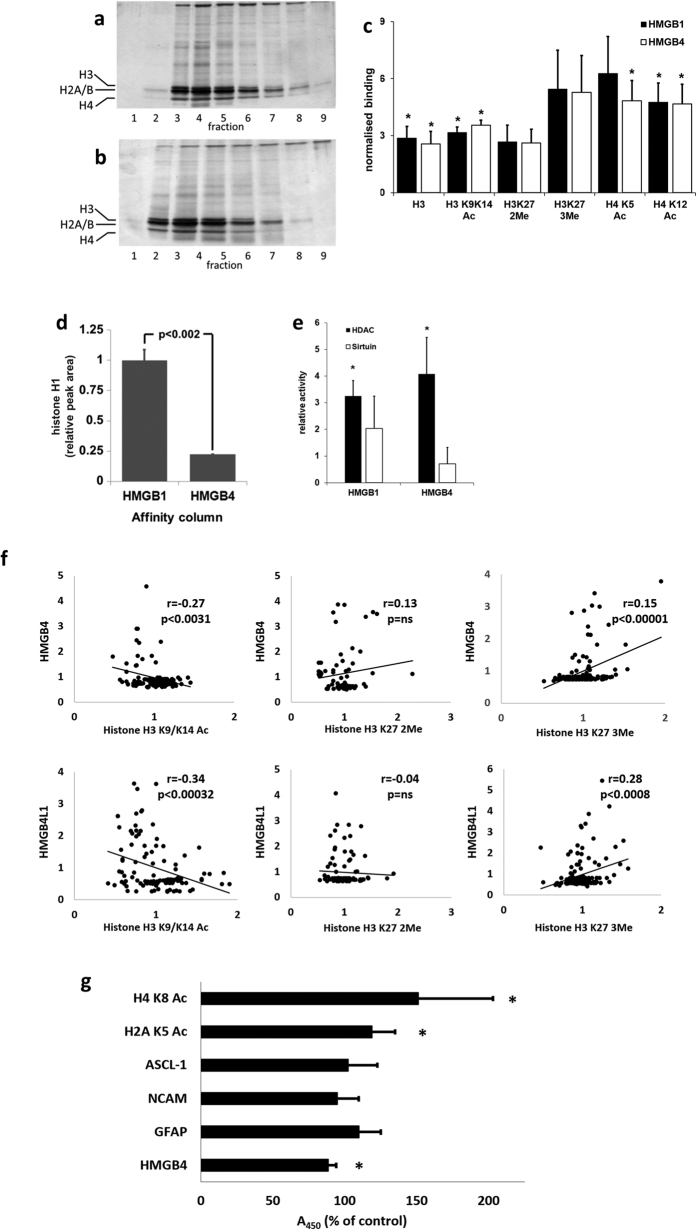
Interactions of HMGB4 and HMGB4L1 with histones. (**a**,**b**) Binding of C6 -cell nuclear proteins to HMGB-protein affinity columns. Proteins in elution fractions were analyzed with silver stained SDS-PAGE. The bands, representing core histones, are indicted with marks on the left. a = HMGB1 –affinity column, b = HMGB4 –affinity column. (**c**) Core histones eluted from HMGB-protein affinity columns and histones were quantified with ELISA. Affinity chromatography was done as described above (n ≥ 3, *p < 0.05, ±SD; Ac = acetylated, 2Me = di-methylated, 3Me = tri-methylated (lysine)). (**d**) HMGB1 and HMGB4 differ in their histone H1 binding capacity. Affinity chromatography was done as described above and eluted proteins were analyzed with histone H1 ELISA. Elution peak areas were quantified (n = 3, ± SD, *p < 0.002). (**e**) HDACs bind to HMGB1 and HMGB4. Nuclear proteins were analyzed with affinity chromatography as described above except that eluted fractions from each column were pooled to form a single fraction. Relative HDAC and sirtuin activities in elution fractions were determined (n ≥ 3 in each experiment, ±SD). (**f** ) Immunofluorescence intensities of modified histone antibody stained HMGB4 or HMGB4L1 overexpressing cells. Rat glioblastoma C6- cells transiently expressing HMGB4-V5 or HMGB4L1-V5 were double-immunostained with anti-acetylated (K9/K14), anti-dimethylated K27 or anti-trimethylated K27 histone H3 antibodies and with anti-V5 antibodies. The correlation blots are shown. (**g**) Regulation of protein levels in neuronal precursor cells by HMGB4 shRNA. Human neuronal precursor NTERA-2 cl. D1 cells stably overexpressing HMGB4 shRNA or control nonspecific shRNA were analyzed in cell ELISA. Absorbance values of HMGB4 shRNA expressing cells were normalized to values of control shRNA expressing cells (n ≥ 3, ± SD, *p < 0.03). H2A K5 Ac = histone H2A acetylated lysine 5, H4 K8 Ac = histone H4 acetylated lysine 8.

STRING database^21^ searches suggested that HMGB4 interacts with transcription regulatory factors, histone H2A and topoisomerase II (Supplementary Table S1). Therefore we next analyzed the role of nuclear proteins in HMGB4 functions. Binding of nuclear proteins, isolated from C6 glioblastoma cells, to HMGB1 and HMGB4 -affinity columns was studied, using a previously described method for recombinant HMGB –protein affinity column liquid chromatography^22^. Core histones bound to both HMGB1- and HMGB4-columns, whereas the linker histone H1 only bound to the HMGB1-column (Fig. 3a–d). The fractions were then analyzed for histone modifying enzymatic histone deacetylase (HDAC) and sirtuin activities. HDAC-activity was significantly increased in HMGB1 and HMGB4 elution fractions whereas sirtuin activity was not (Fig. 3e). Since HMGB4 associated with histones and HDACs we tested whether the transient ectopic expression of HMGB4 or HMGB4L1 in C6 cells or downregulation of endogenous HMGB4 in NTERA2 cl. D1 cells affects the histone post-translational modification. The intensities of anti-acetylated histone H3 K9/K14 and anti-methylated histone H3 K27 staining in HMGB4/HMGB4L1 overexpressing cells were quantified. Acetylated histone H3 K9/K14 and HMGB4/HMGB4L1 stainings were negatively correlated. In contrast, trimethylated histone H3 K27 and HMGB4/HMGB4L1 stainings were positively correlated. There was no correlation between dimethylated histone H3 K27 and HMGB4/HMGB4L1 stainings (Fig. 3f). To monitor the effect of HMGB4 loss on the regulation of protein levels we stably transfected NTERA-2 cl. D1 cells with HMGB4 shRNA. HMGB4 shRNA-expressing cells expressed lower levels of HMGB4 and elevated levels of acetylated histones H2A and H4 (Fig. 3g). Taken together, these results show that both HMGB1 and HMGB4 are associated with core histones and HDACs, and that HMGB4 and HMGB4L1 regulate post-translational modifications of histones in transformed cells derived from the central nervous system.

**Figure 4:**
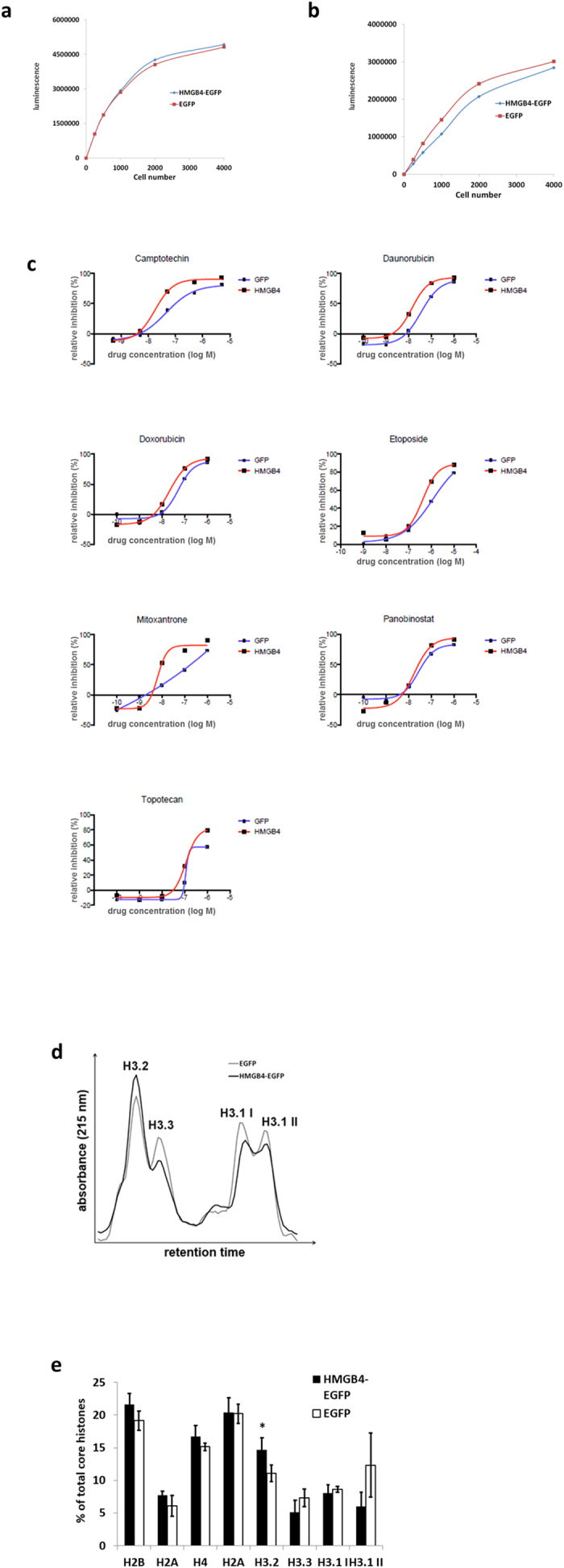
HEK 293T -cells overexpressing HMGB4-EGFP have increased sensitivity to topoisomerase inhibitors and altered histone variant composition. (**a**) Growth of HEK 293T -cell clones with doxycycline induced HMGB4-EGFP or EGFP -expression. The expression of HMGB4-EGFP or EGFP in cells was induced over one week with doxycycline and viability was measured with CellTiterGlo Luminescent assay reagent via ATP quantification. (**b**) Growth of stable HEK 293T -cell clones, constantly expressing HMGB4-EGFP or EGFP. Cells were cultured for 72 h and viability was measured as described above. (**c**) Overexpression of HMGB4 increases cell sensitivity to topoisomerase inhibitors. Cells overexpressing HMGB4-EGFP were more sensitive to topoisomerease inhibitors than control cells expressing EGFP. Figure shows representative curves from two different experiments. (**d**) Quantification of histone H3 variants of HMGB4-EGFP or EGFP-expressing HEK 293T -cell clones. Histones were isolated from the cells and analyzed with RP-HPLC. Histone peaks were identified according to their relative retention times. Histone H3.1 eluted in two peaks (H3.1 I and H3.1 II). Curves are derived from three EGFP -control cell clone analyses and from three HMGB4-EGFP –cell clone analyses. (**e**) Maximal core histone peak heights of RP-HPLC (see above) were determined with the UNICORN- software. Peak height sum of core histones was determined as 100% and relative peak heights were calculated. The relative amount of histone H3.2 was elevated in the HMGB4-EGFP -expressing cells when compared to the control cells (n = 3, ±SD, *p < 0.05).

To further characterize HMGB4 function in somatic cells, doxycycline induced HMGB4-EGFP –overexpressing and constantly HMGB4-EGFP overexpressing HEK 293T -cells were generated through lentivirus infection. Both clones proliferated similarly when compared to EGFP –expressing controls (Fig. 4a,b). Constantly overexpressing HMGB4-EGFP and EGFP expressing cell clones were further analyzed in a drug sensitivity and resistance testing screen with 279 drugs from an oncology collection library^23^ (Institute for Molecular Medicine, Finland). The most promising hits included topoisomerase inhibitors that were selected for further validation. Inhibitors of topoisomerases induced the death of HMGB4 expressing cell cultures at lower concentrations than in control cell cultures (Fig. 4c). These results are consistent with STRING –search results that suggested HMGB4 interactions with topoisomerase 2A and 2B (Supplementary Table S1). Since our STRING search also suggested that HMGB4 interacts with histones (Supplementary Table S1) and HMGB4 associates with core histones (Fig. 3b,c) we analyzed the expression of histones and histone variants in HMGB4-expressing cells. The relative amount of histone H3.2 was elevated in the HMGB4-EGFP -expressing cells when compared to the control cells (Fig. 4d,e). No changes in the composition of the other core histone or the linker histone H1 protein fractions were observed in the RP-HPLC analyses. These results indicate that HMGB4 can induce changes in the sensitivity to topoisomerase inhibitors and in histone composition.

Microarray analysis further indicated that the exogenous expression of HMGB4-EGFP is a strong modulator of transcription since the expression of more than 800 genes with p-values ≤0.013 (n = 3) was altered (Supplementary Table S2). The HMGB4-EGFP construct induced changes in gene expression related to cell adhesion, histones and nucleosomes, vasculature development, cirtullination and processes involving the tumor necrosis factor receptor (Table 1). For example, the expression of many histone variants was significantly changed, including histones H1c, H2ab, H2ad, H2ae and H3d in histone cluster 1, and histone H2ab in histone cluster 2 and H2a in histone cluster 3. In addition, many pro- and anti-apoptotic genes were regulated. However, there was not any clearly increasing or decreasing trend in the expression of apoptosis associating genes in qPCR analysis of HMGB4-EGFP –expressing cells (Supplementary Figure S1).

**Figure 5:**
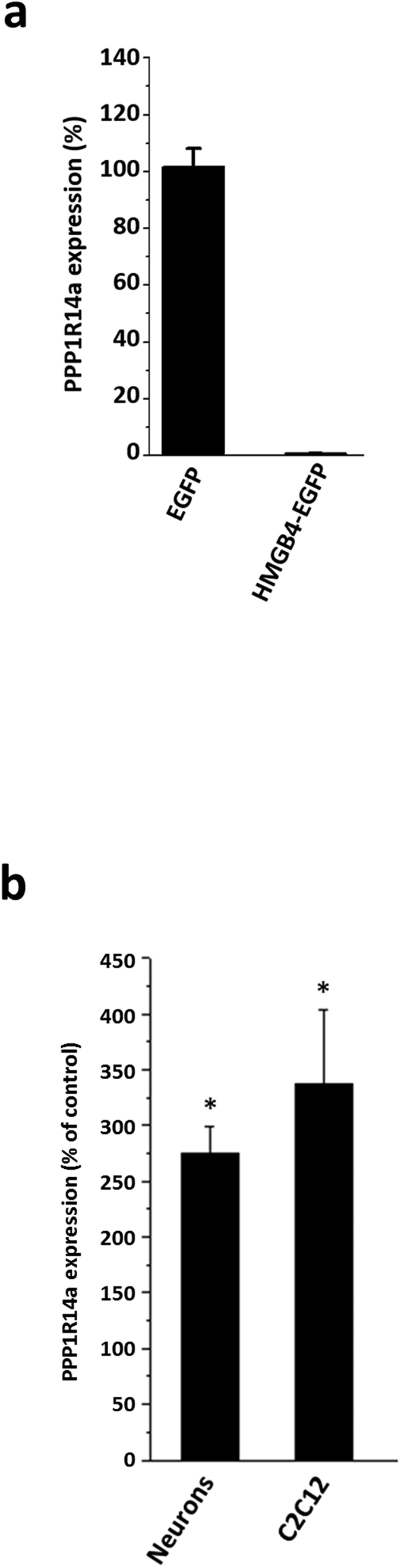
Expression of PPP1R14a is regulated by HMGB4. (**a**) Inhibition of PPP1R14a expression in HEK 293T -cells constitutively expressing HMGB4-EGFP. Three independent repeats were analyzed by comparing representative EGFP expressing control and HMGB4-EGFP expressing cells. Results from representative analysis are shown. (**b**) Mouse E14 cortical neurons and C2C12 myoblast cells, both expressing endogenous HMGB4 mRNA, were treated with HMGB4 *Vivo*-Morpholino to downregulate translation of HMGB4. The expression levels of PPP1R14a were analyzed with qPCR. The levels of the PPP1R14a transcript in HMGB4 *Vivo*-Morpholino -treated cells were normalized to the PPP1R14a transcript levels in control *Vivo*-Morpholino -treated cells (*p < 0.05, ± SEM; n = 6 in C2C12 experiment; n = 18 in neuron experiment).

The most significant change in gene expression, induced by HMGB4-EGFP in microarray analyses was seen in the expression level of the *PPP1R14a* gene, which was downregulated approximately 140 –fold (Supplementary Table S2). *PPP1R14a* codes for protein phosphatase 1 regulatory subunit 14A (also known as C-kinase activated Protein phosphatase-1 Inhibitor of 17 kDa, CPI-17) that regulates multiple cellular functions, including cell adhesion, tumorigenesis and brain development. *PPP1R14a* downregulation in HMGB4-EGFP expressing cell clones was further evaluated by qPCR (Fig. 5a). To confirm that HMGB4 directly regulates the expression of the *PPP1R14a* gene in transformed cells we knocked down endogenous HMGB4 translation in mouse C2C12 myoblast cells (Fig. 5b). When these cells were treated with HMGB4 *Vivo*-Morpholinos, they expressed higher levels of PPP1R14a mRNA than the cells treated with standard control *Vivo*-Morpholinos. As our microarray data revealed, cell adhesion processes were most significantly affected in HMGB4 –overexpressing cells (Table 1). Knock down experiments suggested the possibility that one relevant HMGB4 regulated mechanism in cells is PPP1R14a-dependent regulation of phosphorylation of cell adhesion controlling proteins. One such protein is merlin, when phosphorylated, is a key regulator of adhesion in many cell types^24^,^25^,^26^,^27^. Therefore we tested merlin phosphorylation in HMGB4 overexpressing cells. The relative phosphorylation state of merlin remained unaltered in cell cultures expressing HMGB4-EGFP when compared to control EGFP –expressing cell cultures (0.60 ± 0.31 and 1.00 ± 0.08, respectively). This suggests that other processes than merlin phosphorylation dependent cell adhesion are relevant in HMGB4 mediated regulation of PPP1R14a.

**Figure 6:**
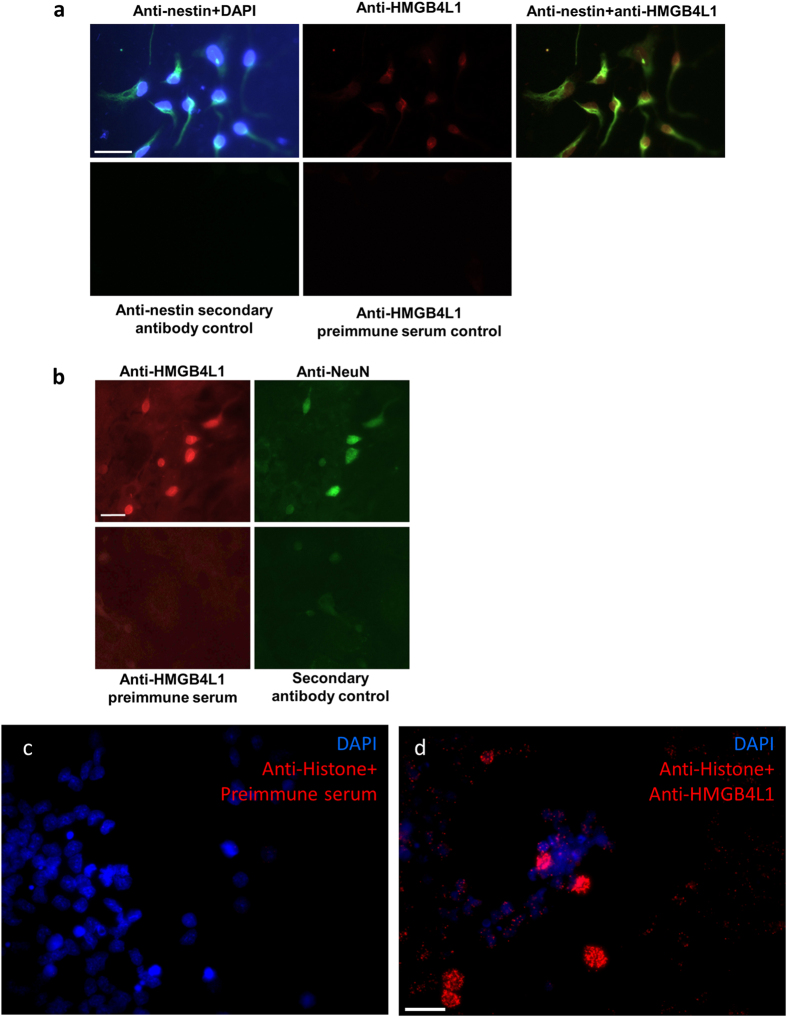
Expression of HMGB4 and HMGB4L1 in cultured primary cells of rat brain. (**a**) Immunofluorescence staining of nestin and HMGB4L1 in 1d differentiated rat neuronal cells *in vitro*. Neurospheres were allowed to adhere and differentiate for 1 day and cells were immunofluorescently stained with anti-nestin and anti-HMGB4L1 antibodies. Cell nuclei were stained with DAPI. The staining controls for anti-nestin or anti-HMGB4L1 staining were done without primary antibody or with preimmune serum, respectively. Scale bar = 30 μm. (**b**) Immunofluorescence staining of HMGB4L1 and NeuN in 14d differentiated rat neuronal cells *in vitro*. Neurospheres were allowed to differentiate for 14 days and cells were immunofluorescently stained with anti-NeuN and anti-HMGB4L1 antibodies. Staining controls for anti-HMGB4L1 and anti-NeuN were done with pre-immune serum or without primary antibody, respectively. Scale bar = 20 μm. (**c**,**d**) Proximity ligation assay of neurospheres with anti-PAN-Histone and anti-HMGB4L1 antibodies. Cell nuclei of cultured neurospheres were stained with DAPI and a proximity ligation assay was performed with anti-PAN-Histone antibodies and preimmune control serum (**c**) or with anti-PAN-Histone antibodies and anti-HMGB4L1 antibodies (**d**). Scale bar = 20 μm.

Since PPP1R14a is a marker of mature oligodendrocytes both in mammals and in zebrafish^28^,^29^ we hypothesized that HMGB4 may have functions in differentiation of brain cells. This hypothesis is further supported by the fact that HMGB4 is expressed in the brain (Supplementary Figure S2) where its expression is associated to extracellular region processes (Table 1). In addition, polymorphisms in the HMGB4 gene locus have been associated with psychiatric diseases^13^,^14^,^15^,^16^. We found mRNA coding for HMGB4 and HMGB4L1 in cultured rat neurons (Fig. 1f). Immunofluorescence staining of rat brain cell cultures with an anti-HMGB4L1 antibody indicated that HMGB4L1 is co-expressed with nestin and neuronal nuclei antigen (NeuN) in neurospheres and in *vitro* differentiated neuronal cells, respectively (Fig. 6a,b). In neurosphere cultures HMGB4L1 co-localizes with histones in the cell nucleus (Fig. 6c,d). Although the expression of HMGB4 decreases during neurosphere differentiation into neurons^5^, our results indicate that differentiated neurons still express some HMGB4/HMGB4L1.

Downregulation of HMGB4 in mouse embryonic neurons by specific *Vivo*-Morpholinos upregulated the expression of PPP1R14a mRNA by 2.75 fold when compared to the expression in neurons treated with standard control *Vivo*-Morpholinos (Fig. 5b). This result and the fact that HMGB4 is downregulated during neurosphere differentiation^5^ suggests that HMGB4 might regulate the development of neuronal tissue. Hence, the effect of endogenous HMGB4 on differentiation of neuronal precursor cells was studied with the well-characterized neuronal precursor cell line model NTERA-2 cl. D1 that expresses HMGB4 (Fig. 3g and ref. 30). To monitor the effect of HMGB4 loss on the regulation of neuronal differentiation-related gene expression we stably transfected NTERA-2 cl. D1 cells with HMGB4 shRNA to down-regulate the expression of HMGB4, and we conducted a qPCR-array. The array revealed a large number of genes to be affected (Table 2). Downregulation of HMGB4 in NTERA-2 cl. D1 cells, for instance, upregulated the gene expression of the neuroblast formation marker, ASCL1, and downregulated the mature neuron and astrocyte gene expression markers, FABP7, CNP, MBP, NR4A2 and NEUROD1. Further, downregulation of HMGB4 decreased the expression of the neuronal cell adhesion and maturation molecule, NCAM1 -coding gene (Table 2). Taken together, these results indicate that HMGB4 regulates the expression of genes involved in maturation and differentiation of neuronal precursor cells.

## Discussion

In this study, we show that HMGB4 and the highly similar HMGB4L1, regulate chromatin and differ in many aspects from HMGB1, the archetype member in the family of HMGB proteins. Furthermore, according to our knowledge, this is the first study elucidating the functions of endogenous HMGB4 in somatic cells showing regulation of gene expression by HMGB4.

Human and mouse genomes have a single *HMGB4* gene whereas in rat, in addition to the *HMGB4* gene on chromosome 5, a similar *HMGB4L1* gene exists on chromosome X. Rat, mouse and human *HMGB4* genes have a similar genomic organization within an intron of the *CSMD2* gene^31^, which clearly differs from the genomic organization of the *HMGB4L1* gene that is localized to rat X -chromosome. The transcripts HMGB4 and HMGB4L1 are, however, similar suggesting that the *HMGB4L1* gene originates from the *HMGB4* gene. Furthermore, similar expression patterns in rat cells and tissues and similar behavior in cells, suggest a close relationship between the two proteins.

Among species, HMGB1, -2 and -3 intron - exon structures and coding sequences are highly conserved^32^. In this respect the HMGB4 and HMGB4L1 genes seem to differ from other HMGB genes. The coding sequences are less conserved between rat HMGB4 and HMGB4L1. Further, the HMGB4 gene is less conserved across different species than the HMGB1, -2 or -3 genes. Whereas human HMGB1, -2 or -3 have hardly any amino acid changing mutations, human HMGB4 has a non-synonymous SNP (Fig. 2b).

Our results suggest that HMGB4 and HMGB4L1 may influence chromatin structure. HMGB4 increased the relative expression of the histone H3.2 that has been shown to contain silencing modifications and to associate with facultative heterochromatin^33^,^34^. In addition, HMGB4 associated with HDACs, reduced the acetylation level of the lysine residues of histones H2A, H3 and H4 and increased the level of trimethylation of lysine 27 of histone H3. Also HMGB4L1 was found to reduce the acetylation level of the lysine residues of histone H3 and increase level of trimethylation of lysine 27 of histone H3.These findings suggest a role for post-translational modifications of histones in the regulation of transcription by HMGB4. In addition, overexpression of HMGB4 made cells more susceptible to death by topoisomerase inhibitors suggesting that topoisomerase-related mechanisms are mediating the survival of HMGB4 -expressing cells.

The acidic tail that mediates the binding of HMGB1 to histone H1 is lacking in HMGB4 and may explain the poor binding of HMGB4 to histone H1^35^. In FRAP-analyses, HMGB4, HMGB4L1 and an acidic tail- deleted form of HMGB1, resembling the domain structure of HMGB4 and HMGB4L1, show lower mobility in the nucleus compared to the full-length form of HMGB1^19^,^20^. Furthermore, truncated HMGB2 lacking an acidic tail binds to chromosomes more strongly than the full-length form of HMGB2^36^. Thus, the acidic tail seems to weaken the association of HMGB -proteins to nuclear structures.

Microarray analyses of HMGB1 -deleted cells and tissues have revealed some changes in gene expression^37^,^38^. In this study, significant changes in gene expression were seen in neuronal precursor cells in which HMGB4 was knocked down. In addition, the ectopic overexpression of HMGB4 in transformed cells caused drastic changes in the expression of hundreds of genes, indicating the potency of HMGB4 to regulate transcription. The pronounced effects of HMGB4 on transcription are consistent with its strong binding to the nucleus, interactions with histones and regulation of histone variant expression.

Most somatic cell types do not express HMGB4 or express it at only very low levels. Exceptions are neurospheres and neurons and we hypothesized that HMGB4 might regulate transcription in neuronal cells. Our results show that inhibition of endogenous HMGB4 in cells downregulated markers of mature neurons. The oligodendrocyte maturation marker PPP1R14a was strongly downregulated by HMGB4 and upregulated in cells with downregulated endogenous HMGB4 expression^28^,^29^. PPP1R14a gene expression is regulated by various factors including NeuroG2, Hoxb1b (a zebrafish Hoxa1 homolog) and SP1/3^39–41^. The zebrafish PPP1R14a homolog PPP1R14al regulates both early and late brain development in the embryonal stage suggesting that HMGB4 may regulate brain development via a PPP1R14a mediated mechanism in mammals^40^.

Inhibition of endogenous HMGB4 expression in neural progenitor cells caused most prominently the downregulation of the Neuro D1 gene (Table 2), a potent neural differentiation factor^42^. The downregulation of this transcription factor would be consistent with the view that HMGB4 regulates neural differentiation by modulating gene expression in neural cells. The finding that expression of several neuronal and glial genes is downregulated together with Neuro D1 (Table 1) is also consistent with this view.

In contrast, the ASCL1 gene was found to be upregulated upon inhibition of endogenous HMGB4 (Table 2). ASCL1 is a transcription factor essential for the formation of neuroblasts, proliferating precursors of neurons^43^. It appears that downregulation of HMGB4 expression in neuronal precursors allows cells to advance to the neuroblast stage. Expression of HMGB4 in mature neurons may be then required to maintain and modify the neuronal phenotypes during adult neuronal plasticity.

In conclusion, HMGB4 differs from other mammalian members of the HMGB-family. It binds strongly to nuclear structures, regulates chromatin and mediates differentiation of neuronal cells. Strong effects on transcription, and regulation of different histone forms likely underlie the prominent effects of HMGB4 on cell differentiation.

## Materials and Methods

### Sequence analyses

Homology searches of nucleotide databases and SNP-analyses were done using tools on the websites of the Center for Biotechnology Information (Rockville Pike, Bethesda, Maryland, USA) and Ensembl (EMBL-EBI and the Sanger Institute, Cambridgeshire, United Kingdom). Sequence alignments were done using ClustalW on the EMBnet website (The ISREC Bioinformatics Groups, Epalinges, Switzerland). Protein domains were detected using the Pfam program (Wellcome Trust Sanger Institute, Cambridge, UK.). The human HMGB4 isoform was detected in genomic DNA isolated from cultured cells and amplified with primers atgggaaaagaaatccagcta and ggatccatcagctctgcctg. The amplified DNA was analyzed with Ban 1 restriction digestion to detect rs10379 SNP that lacks the Ban 1 cleavage site. Gene expression data of human brain areas was obtained from Medisapiens Ltd. (Medisapiens Ltd, Helsinki, Finland). Gene clusters associating with HMGB4 were analyzed with PANTHER –software^44^.

### Recombinant protein Western blot

Recombinant mouse HMGB4 was produced in HMGB4 cDNA -containing pGEX-6P-1 plasmid-transformed bacteria using previously described methods^45^. Recombinant HMGB1 was produced as described^46^. Recombinant proteins were analyzed in anti-HMGB4 (Abcam, Cambridge, UK) and anti-HMGB4L1 (anti-TP4)^7^ Western blot.

### Northern blot

Total RNA was isolated from laboratory mouse and rat organs, and electrophoresed using agarose gels. Probes were produced from the entire coding sequence of mouse HMGB4 or rat HMGB4L1 cDNAs by PCR. RNA was blotted onto nylon filters, blocked, and probed with ^32^P-labelled probes using conventional Northern-blotting methods.

### Immunohistochemistry

4% PFA-fixed, paraffin embedded tissue sections of adult rat testis were deparaffinized in a decreasing series of alcohol. The antigen was unmasked by heating the sections in 10 mM Na-citrate buffer for 15 min in a microwave oven. The sections were then rinsed in PBS and endogenous peroxidase was blocked by incubating the sections in 3% H_2_O_2_ in PBS. Unspecific binding sites were blocked by incubation in 5% fat-free milk powder in PBS for 1 h at room temperature. The anti-HMGB4L1 antibody was then applied in a 1:50 dilution in blocking solution and the sections were incubated at 4 °C overnight. The primary antibody was washed off with PBS and the sections were incubated for 1 h at room temperature in a 1:300 dilution of anti-rabbit-HRP antibody (GE Healthcare, Pittsburgh, PA, USA) in blocking solution. The antigen was detected by incubating the sections in aminoethylcarbazole substrate (Sigma-Aldrich, St. Louis, MO, USA). Nuclei were visualized by counterstaining in haematoxylin.

### *In Situ* Hybridization

Sequences for antisense and sense probes were obtained from Allen Brain Atlas (Allen Institute for Brain Science, Seattle, WA, USA) and digoxigenin-labelled RNA probes were produced. Paraffin-embedded sections of adult mouse testis were deparaffinized, washed and digested and permeabilized with proteinase K and Triton X-100. Sections were washed and refixed in 4% PFA. After washing, sections were hybridized with either antisense or sense probes at 65 °C overnight, washed and blocked with 2% sheep serum, 2mg/ml BSA in PBS/Triton X-100. The sections were then incubated in anti-digoxigenin-AP antibody (Roche, Mannheim, Germany) 1:2000 in blocking solution at 4 °C overnight, washed and detected in alkaline phosphatase buffer with nitro-blue tetrazolium and 5-bromo-4-chloro-3′-indolyphosphate (Sigma-Aldrich) as substrates in the dark.

### Cells and tissues

Rat hippocampal neurons were isolated and cultured as described^47^. Rat cerebellar neurons were isolated from young rats, and cultured in DMEM supplemented with 10% FCS, 2 mM glutamine, 25 mM KCl, penicillin and streptomycin. Rat neurospheres were isolated as described and cultivated in NeuroCult NSC Basal Medium containing Proliferation Supplements (StemCell Technologies, Vancouver, Canada)^48^. Mouse neurons were isolated from E14 mouse cerebral hemispheres as described^48^.

### RT-PCR analysis of gene expression

Total RNA was isolated from rat neurons using RNeasy kit (Qiagen, Valencia, CA, USA) and treated with DNAse I (Roche), and reverse transcribed using M-MLV reverse transcriptase (Promega, Madison, VI, USA). DNA was amplified using DynaZyme DNA-polymerase (Finnzymes, Espoo, Finland) using oligos atgggaaaagacagcaaagtaagacc and ttactccccgcgctggcatt for rat HMGB4L1, or atggggaaaaaagtccagctaagac and ttattctttcaaactgcttttggacg for rat HMGB4. Resulting products were analysed in agarose gel electrophoresis.

### Immunofluorescence of modified histone H3 proteins

Rat HMGB4 and HMGB4L1 cDNAs were cloned into pcDNA6 plasmids (Life Technologies, Carlsbad, CA, USA). C6 -cells were transfected with the plasmids and cultured overnight to allow for the expression of V5-tagged proteins. The cells were immunofluorescently stained with anti-V5-tag (Thermo Fisher Scientific Inc., Rockford, IL, USA) and with anti-modified histone H3 antibodies (anti-histone H3 acetylated K9/K14, anti-histone H3 di-methylated K27 and anti-histone H3 tri-methylated K27; Millipore, Billerica, MA, USA) to identify HMGB4- and HMGB4L1- expressing cells and to detect modified histones. Digital pictures were taken and analyzed with ImageJ software^49^.

### Cloning of EGFP-fusion proteins

cDNA coding for mouse HMGB4 was cloned to a pEGFP-N1 vector (ClontechTakara Bio Company, Shiga, Japan). Mouse HMGB1-EGFP and an empty EGFP-vector served as controls. Rat HMGB4L1, human HMGB4 and human HMGB4 rs10379 SNP -variant were cloned to a pEGFP-C1 vector (ClontechTakara Bio Company). HMGB4-EGFP- and EGFP- coding lentiviruses were produced using a method described earlier^50^ and they were used to produce stable doxycycline-inducible or constantly HMGB4-EGFP or EGFP –overexpressing HEK 293T -cells.

### FRAP analyses

NIH-3T3 cells were transiently transfected with EGFP-tagged proteins. Confocal imaging was performed using a Leica SP2 AOBS confocal microscope (Leica Microsystems GmbH, Wetzlar, Germany) at the Light Microscopy Unit of the Institute of Biotechnology, University of Helsinki. For comparing the mobility of HMGB1 (n = 6), HMGB4 (n = 4) and HMGB4L1 (n = 5) three pre-bleach images were taken followed by two bleach pulses at 488 nm covering half of the nucleus. Images were collected at different time points after bleaching. To compare the effects of SNP on the mobility of HMGB4, five pre-bleach images were taken followed by three bleach pulses at 488 nm, quenching one brightly fluorescent area/nucleus. Post-bleach -images were collected at different time points.

The fluorescence images were analyzed using Leica Confocal software (Leica Microsystems GmbH). Recovery curves were generated without subtracting the background, and t_1/2_ was calculated as described^51^.

### Affinity chromatography analyses

Recombinant HMGB1 and HMGB4 were coupled to Sepharose-affinity column and affinity chromatography of C6 –cell nuclear proteins was done as described^22^. Histone contents in flow through and eluted protein fractions were analyzed with SDS-PAGE and histone-ELISA using previously described methods^22^. HDAC -activity in the starting material, in flow through and in eluted protein fractions was analyzed with substrate Ac-Arg-Gly-Lys(Ac)-AMC (Anaspec, Fremont, CA, USA) as described^52^. Sirtuin -activity was measured using the same assay with additional 1 mM nicotinamide adenine dinucleotide (Sigma-Aldrich) and 600 nM trichostatin A (Sigma-Aldrich) in the assay buffer. The protein content in the samples was analyzed with the BioRad protein assay (Biorad, Hercules, CA, USA).

### Gene and protein expression analyses of NTERA-2 cl.D1 cells

NTERA-2 cl. D1 cell were obtained from the American Type Culture Collection (Manassas, VA, USA). Human HMGB4 shRNA was purchased from Fisher Scientific (Pittsburgh, PA, USA). The control shRNA was produced as described^50^. Cells were infected with lenti-viruses and positive cells were selected after puromycin treatment^50^. RNA was isolated from the cells, reverse transcribed and analyzed with qBiomarker Validation PCR Array Neuronal Differentiation (Qiagen). The expression level of HMGB4 was analyzed in separate qPCR with specific primers. Protein levels in cells were measured with cell ELISA as described previously^53^. The following antibodies were used in cell ELISA: mouse anti-β-Actin (clone AC-15; Sigma-Aldrich), mouse anti-GFAP (clone G-A-5; Sigma-Aldrich), mouse anti-NCAM (clone 5B8; Developmental Studies Hybridoma Bank, University of Iowa, Iowa City, IA, USA), rabbit anti-ASCL1 (Biobryt Ltd., Cambridgeshire, UK), rabbit anti-human HMGB4 (Boster, Pleasanton, CA, USA), rabbit anti-histone H2A acetylated Lys5 and rabbit anti-histone H4 acetylated Lys8 (Cell Signaling Technology, Danvers, MA, USA).

### Stable cells expressing HMGB4-EGFP and EGFP

HEK 293T -cells were infected with lenti-viruses coding for HMGB-EGFP or EGFP fusion proteins and stably over-expressing clones were obtained from single cells.

### Drug sensitivity and resistance testing screen

Stable HEK 293T –cells constantly over-expressing HMGB-EGFP or control EGFP were seeded in a density of 1000 cells per well in 384 -well plates. 279 compounds from the FIMM (Institute for Molecular Medicine Finland, Helsinki, Finland) oncology collection library were added in different concentrations. After 72 h of incubation (at 37 °C; 5% CO_2_), cell viability was measured by CellTiterGlo Luminescent assay (Promega). The screen was described in details earlier^23^.

### Microarray analyses of HMGB4-EGFP over-expressing HEK 293T -cell clones

Human embryonic kidney HEK 293T -cells stably over-expressing HMGB-EGFP or control EGFP were cultured to the sub- confluent stage and RNA was isolated with Trizol (Thermo Fisher Scientific Inc.). aRNA was amplified with Amino Allyl MessageAmp II aRNA Amplification Kit (Thermo Fisher Life Technologies, Waltham, MA, USA) and labeled with CyDye Post-Labelling Reactive Dye (GE Healthcare).

Unrestricted Human Genome Microarray 4x44K microarray slides (Agilent, Santa Clara, CA, USA) hybridized with HEK 293T –cell-derived labelled aRNA were scanned by an Axon GenePix 4200 AL (Molecular Devices, Downington, PA, USA) scanner and median intensity of each spot was estimated using GenePix Pro 6.0 software (Molecular Devices, Sunnyvale, CA, USA). The data were analyzed with R and Bioconductor package Limma^54^. The linear model and empirical Bayesian methods were applied to find differentially expressed genes (p-value <0.01). Function annotation clustering of differentially expressed genes was analyzed in DAVID^55^.

### RP-HPLC analyses of histone variants

Histones from stable HEK 293T -cell clones overexpressing HMGB4-EGFP or EGFP were acid-extracted as described^56^. Histone RP-HPLC with acetonitrile buffers as a mobile phase was done as described using ÄKTA Micro RP-HPLC equipped with a Resource 5 RPC column (GE Healthcare)^56^.

### *Vivo*-Morpholino -induced translational inhibition

Mouse PPP1R14a expression levels in E14 mouse cortical neurons treated with *Vivo*-Morpholinos (Gene Tools LLC, Philomath, OR, USA) for 6 days and C2C12 -cells treated with *Vivo*-Morpholinos for 3 days were analyzed with qPCR. Morpholino sequences were 5′ tggtctttttcccccatgtttatac-3′ (mouse HMGB4 start) and 5′-cctcttacctcagttacaatttata-3′ (standard control). Morpholinos were added to cells in a 1/800 dilution.

### Analysis of merlin

Phosphorylated merlin Western –blots were done as described previously^24^.

### Immunofluorescence staining of neurospheres and neurons

Rat neurospheres were isolated as described and cells were allowed to adhere and differentiate for 1 to 14 d^48^. Subsequently, cells were immunofluorescently stained with anti-nestin (Developmental Studies Hybridoma Bank), anti-HMGB4L1 or anti-NeuN (Millipore) antibodies. Cell nuclei were stained with 4′,6-diamidino-2-phenylindole (DAPI; Sigma-Aldrich). Controls for nestin or HMGB4L1 stainings were performed without the primary antibody or with preimmune serum. A proximity ligation assay of neurospheres with anti-PAN-Histone (Millipore), anti-HMGB4L1 antibodies and preimmune control serum was done using Duolink Kit (Olink Bioscience, Uppsala, Sweden).

### Ethics statement

The animal experiment permits were obtained from the Office of the Regional Government of Southern Finland (license number ESAVI/6603/04.10.03/2011) in agreement with the ethical guidelines of the European convention. All experimental protocols were carried out in accordance with the approved guidelines of the Laboratory Animal Centre (LAC) of the University of Helsinki.

### Statistical analysis

qPCR data was examined as described^57^. Other values were calculated with Excel (Microsoft Corporation, Redmond, WA) and are expressed as means ± SD. The data were examined using Student’s t-test or Pearson correlation analyses to calculate p-values. For all statistical tests, significance was accepted at p < 0.05.

## Additional Information

**How to cite this article**: Rouhiainen, A. *et al*. HMGB4 is expressed by neuronal cells and affects the expression of genes involved in neural differentiation. *Sci. Rep.* **6**, 32960; doi: 10.1038/srep32960 (2016).

## Supplementary Material

Supplementary Information

Supplementary Information

## Figures and Tables

**Table 1:** Biological processes associated with HMGB4 expression.

Term	Count	%	p-value	FDR
**293T cells HMGB4-EGFP overexpression**				
GO:0007155 ~ cell adhesion	44	7.82	0.000002	0.003
GO:0022610 ~ biological adhesion	44	7.82	0.000002	0.003
PIRSF002048:histone H2A	7	1.24	0.000003	0.004
GO:0001568 ~ blood vessel development	22	3.91	0.000007	0.013
IPR002119:Histone H2A	7	1.24	0.000009	0.014
GO:0000786 ~ nucleosome	11	1.95	0.000012	0.016
GO:0001944 ~ vasculature development	22	3.91	0.000011	0.019
SM00414:H2A	7	1.24	0.000022	0.027
citrullination	7	1.24	0.000021	0.030
IPR007125:Histone core	9	1.60	0.000030	0.046
repeat:TNFR-Cys 3	7	1.24	0.000029	0.048
**Human brain endogenous HMGB4**				
GO:0005576 ~ extracellular region	73	16.01	0.000030	0.040

HMGB4-EGFP and EGFP -expressing HEK 293T -cell clones were subjected to microarray gene expression analysis. Expression data of human brain tissue samples was obtained from Medisapiens Ltd. Expression data was analyzed with the PANTHER software to detect changes in the gene expression related to various biological processes. Percent of gene hits against total were calculated. Clusters and subclusters with highest values are shown in detail.

**Table 2:** Neuronal differentiation qPCR-array of HMGB4 shRNA -infected NTERA-2 cl. D1 cells.

	GENE	CELL TYPE/FUNCTION	AVERAGE (±SE MEAN)	P
DOWNREGULATED	NEUROD1	TRANSCRIPTION FACTOR ENHANCING NEURONAL DIFFERENTIATION	0.071 ± 0.017	0.014
	MBP	OLIGODENDROCYTE	0.114 ± 0.026	0.000001
	FABP7	ASSOCIATED TO RADIAL GLIA AND NEUROGENESIS	0.123 ± 0.025	0.000001
	SLC32A1	GABA NEURON	0.132 ± 0.042	0.01
	HMGB4	NEURON	0.237 ± 0.052	0.0001
	NR4A2	DOPAMINERGIC NEURON	0.301 ± 0.073	0.003
	NCAM1	MATURE NEURON	0.357 ± 0.084	0.035
	GFAP	ASTROCYTE	0.377 ± 0.087	0.012
	CNP	OLIGODENDROCYTE	0.606 ± 0.011	0.011
UNREGULATED	MAPT	NEURON	0.327 ± 0.071	0.15
	S100B	MATURE NEURON, ASTROCYTE	0.438 ± 0.12	0.13
	PAX6	RETINAL PROGENITOR	0.449 ± 0.094	0.19
	GAD2	GABA NEURON	0.478 ± 0.15	0.15
	FOXG1	MOTONEURON	0.626 ± 0.19	0.21
	OLIG2	OLIGODENDROCYTE PROGENITOR	1.035 ± 0.22	0.92
	NKX2-2	OLIGODENDROCYTE PROGENITOR, OLIGODENDROCYTE	1.052 ± 0.17	0.85
	SLC1A2	GLUTAMATERGIC NEURON	1.118 ± 0.31	0.80
	SLC1A3	GLUTAMATERGIC NEURON	1.217 ± 0.24	0.55
	EMX1	NEURON	1.644 ± 0.44	0.26
UPREGULATED	ASCL1	NEUROBLAST FORMATION	3.917 ± 1.40	0.01

Gene expression was analyzed with neuronal differentiation qPCR array and with HMGB4 qPCR, and the expression levels of HMGB4 shRNA -infected cells were normalized to the expression levels of control shRNA -infected cells. Average fold changes in gene expression are shown (n = 4 in neuronal differentiation qPCR array, and in HMGB4 analysis n = 3 for controls and n = 12 for HMGB4).

One-way ANOVA was used to calculate p-values.
