# Supplementary material for: HMGB4 is expressed by neuronal cells and affects the expression of genes involved in neural differentiation
Source: Sci Rep. 2016 Sep 9;6:32960. doi: 10.1038/srep32960 (PMC5036535; doi:10.1038/srep32960)
Supplement: Supplementary Information [file srep32960-s1.pdf]

Supplementary information

HMGB4 is expressed by neuronal cells and affects the expression of genes involved in neural differentiation

Ari Rouhiainen, Xiang Zhao, Päivi Vanttola, Kui Qian, Evgeny Kulesskiy, Juha Kuja-Panula, Kathleen Gransalke, Mikaela Grönholm, Emmanual Unni, Marvin Meistrich, Li Tian, Petri Auvinen and Heikki Rauvala

Supplementary figure S1. qPCR analysis of pro-apoptotic gene expression in HMGB4-EGFP –expressing cells. H1, H2A and H3A = HMGB4-EGFP expressing cells; E3, E4 and E5 = EGFP –expressing cells.

Supplementary figure S2. Expression of HMGB4 in human brain tissue samples. Human brain expression data was obtained from Medisapiens –database and HMGB4 expression in different brain areas was analyzed with PANTHER -software.

Supplementary table S1. STRING database search predicted HMGB4 protein interaction partners. Different mammalian species were included in the search. The six most prominent hits are shown. SUPT16H = Facilitates chromatin transcription complex subunit suppressor of Ty 16 homolog; SSRP1 = Structure specific recognition protein 1 ; TOP2A/B = Topoisomerases 2A and 2B ; H2AFV/Z = Histone H2A.V and H2A.Z ; GFT2A1L/B = general transcription factor IIA, 1-like and general transcription factor IIB ; POLR1E = DNA-directed RNA polymerase I subunit RPA49.

Supplementary table S2. List of genes differentially expressed in HMGB4-EGFP expressing HEK 293T-cells.

Supplementary figure S1

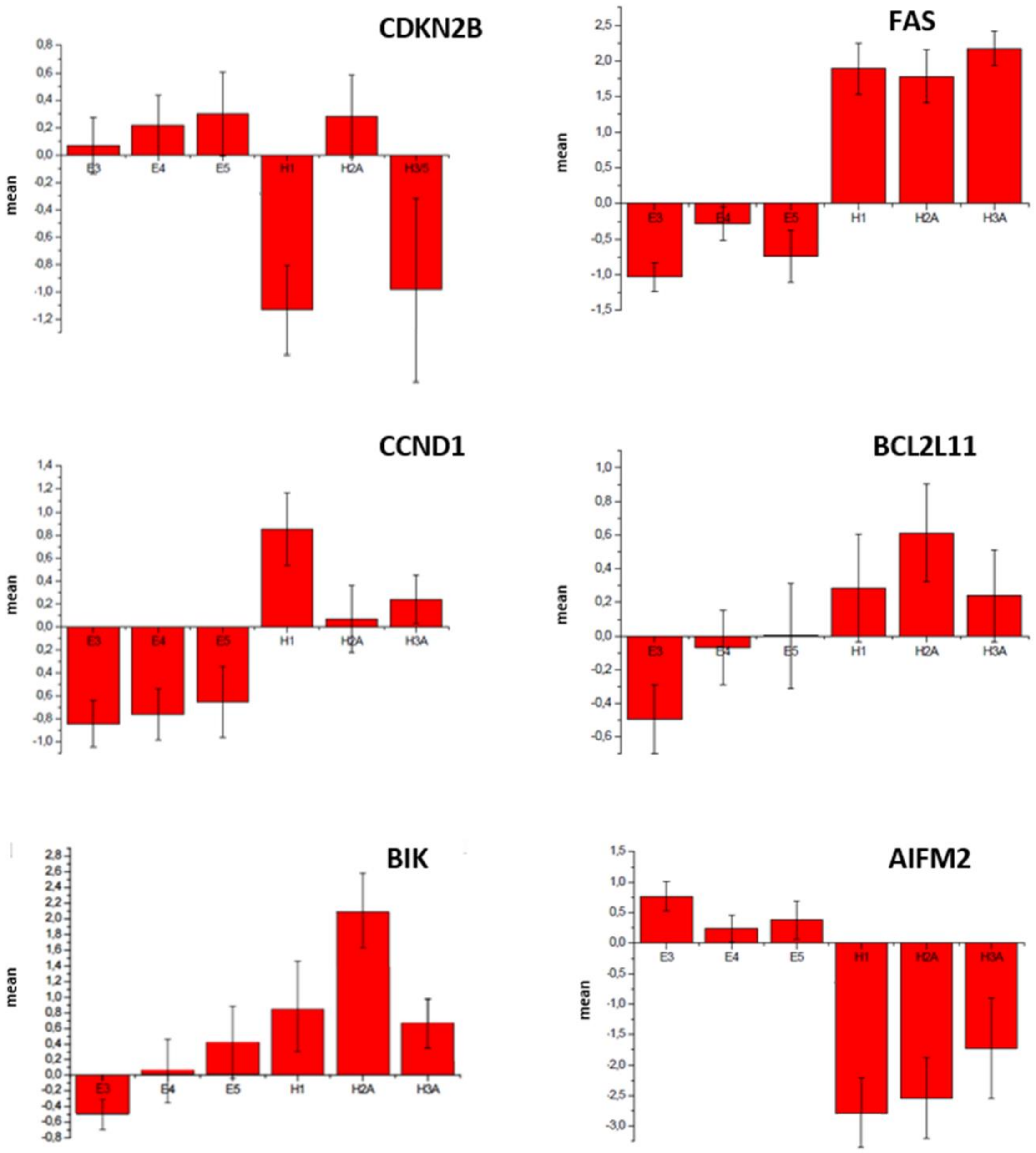

Supplementary figure S2

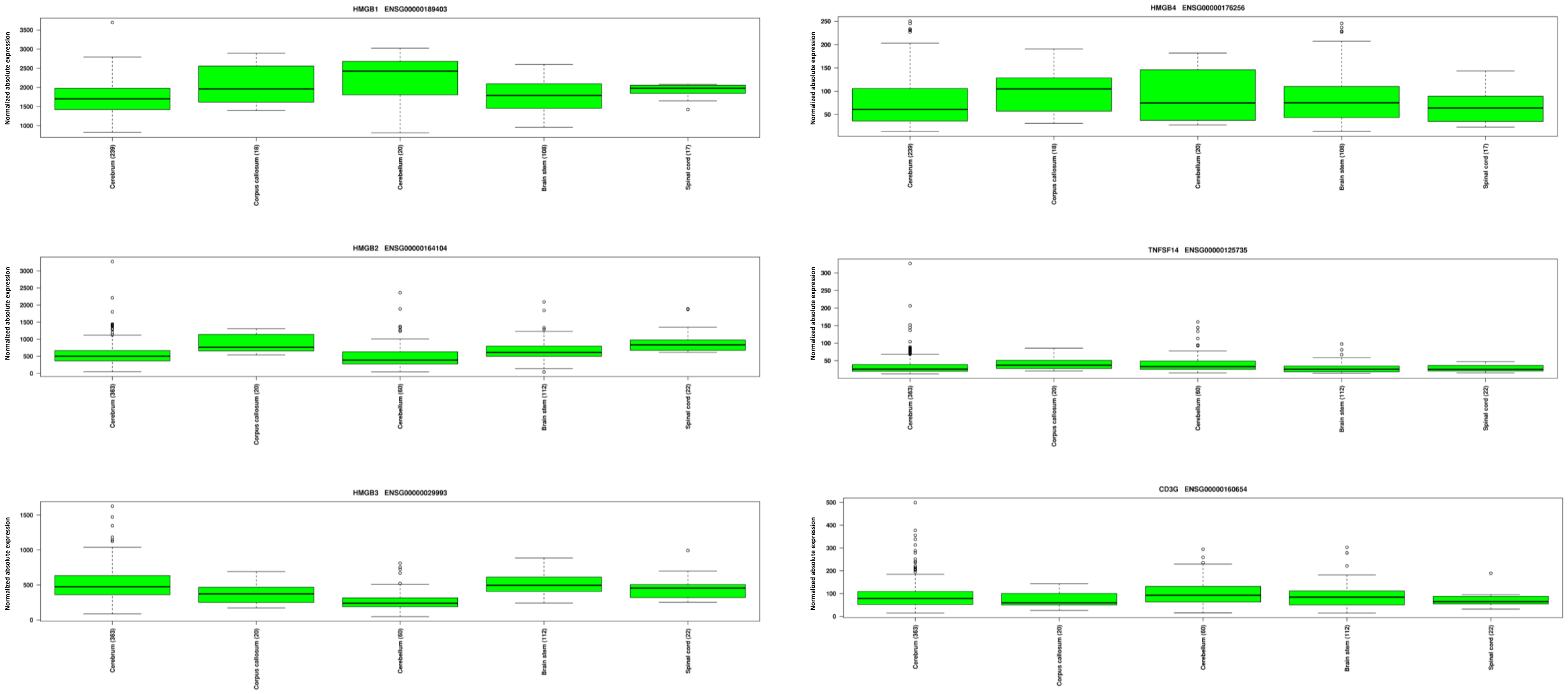

Supplementary table S1

	SUPT16H	SSRP1	TOP2A/B	H2AFV/Z	GFT2A1L/B	POLR1E
**Homo sapiens**	v	v	v	v		
**Mus musculus**	v	v			v	
**Rattus norwegius**	v	v			v	
**Bos taurus**	v		v		v	v
**Monodelphins domestica**	v		v	v	v	v
**Dasypus novemcinctus**	v	v				v
**Pteropus vampyrus**	v	v		v	v	v
**Sarcophilus harrisii**	v		v	v	v	v
**Tursiops truncatus**	v		v	v		v
